# A feasibility study of colorectal cancer diagnosis via circulating tumor DNA derived CNV detection

**DOI:** 10.1371/journal.pone.0196826

**Published:** 2018-05-23

**Authors:** Bhuvan Molparia, Glenn Oliveira, Jennifer L. Wagner, Emily G. Spencer, Ali Torkamani

**Affiliations:** 1 The Scripps Translational Science Institute, La Jolla, CA, United States of America; 2 The Department of Integrative Structural and Computational Biology, The Scripps Research Institute, La Jolla, CA, United States of America; 3 The Department of Molecular and Experimental Medicine, The Scripps Research Institute, La Jolla, CA, United States of America; 4 Scripps Health, La Jolla, CA, United States of America; CNR, ITALY

## Abstract

Circulating tumor DNA (ctDNA) has shown great promise as a biomarker for early detection of cancer. However, due to the low abundance of ctDNA, especially at early stages, it is hard to detect at high accuracies while keeping sequencing costs low. Here we present a pilot stage study to detect large scale somatic copy numbers variations (CNVs), which contribute more molecules to ctDNA signal compared to point mutations, via cell free DNA sequencing. We show that it is possible to detect somatic CNVs in early stage colorectal cancer (CRC) patients and subsequently discriminate them from normal patients. With 25 normal and 24 CRC samples, we achieve 100% specificity (lower bound confidence interval: 86%) and ~79% sensitivity (95% confidence interval: 63% - 95%,), though the performance should be considered with caution given the limited sample size. We report a lack of concordance between the CNVs detected via cfDNA sequencing and CNVs identified in parent tissue samples. However, recent findings suggest that a lack of concordance is expected for CNVs in CRC because of their sub-clonal nature. Finally, the CNVs we detect very likely contribute to cancer progression as they lie in functionally important regions, and have been shown to be associated with CRC specifically. This study paves the path for a larger scale exploration of the potential of CNV detection for both diagnoses and prognoses of cancer.

## Introduction

Circulating cell free DNA (cfDNA) has been recently the focus of study as a promising new biomarker for infectious diseases [1], prenatal testing [2], and cancer [3]. Five-year survival rates for cancer are the highest when cancer is detected early, but efficient and highly specific means to detect cancer early are lacking. Unlike currently used correlative biomarkers, circulating tumor DNA (ctDNA) signatures, a by-product of cancer cell death, can be a causal, and thus more specific biomarker [4]. However, ctDNA abundance can be as low as <1% of all cfDNA. This poses a challenge for the development of a widely available and economical ctDNA based early detection method for cancer. Recently, novel methods have shown success in this endeavor by using either targeted highly sensitive methods to detect point mutations [5] or a combination of targeted methods and protein biomarkers [6]. As compared to point mutations, somatic copy number variants (CNVs) can be several megabases long and contribute a much larger number of tumor-derived DNA molecules to total cfDNA. We have recently shown, *in silico*, that large (>100Mb) somatic CNVs can be used to predict the presence and type of cancer, and, theoretically, that we should also be able to detect them at a relatively low sequencing depth [7]. The potential for cancer screening via detection of tumor-derived CNVs in cfDNA has been demonstrated, to some extent, by prior studies, but not extensively for early-stage tumors which are the major target of interest for cancer screening [8]. To explore this further, we designed a pilot scale study where we collected blood and tissue samples from colorectal cancer patients at various, but mostly early, stages of their diseases and attempted to detect tumor derived CNVs from cfDNA.

## Results

**Fig 1:**
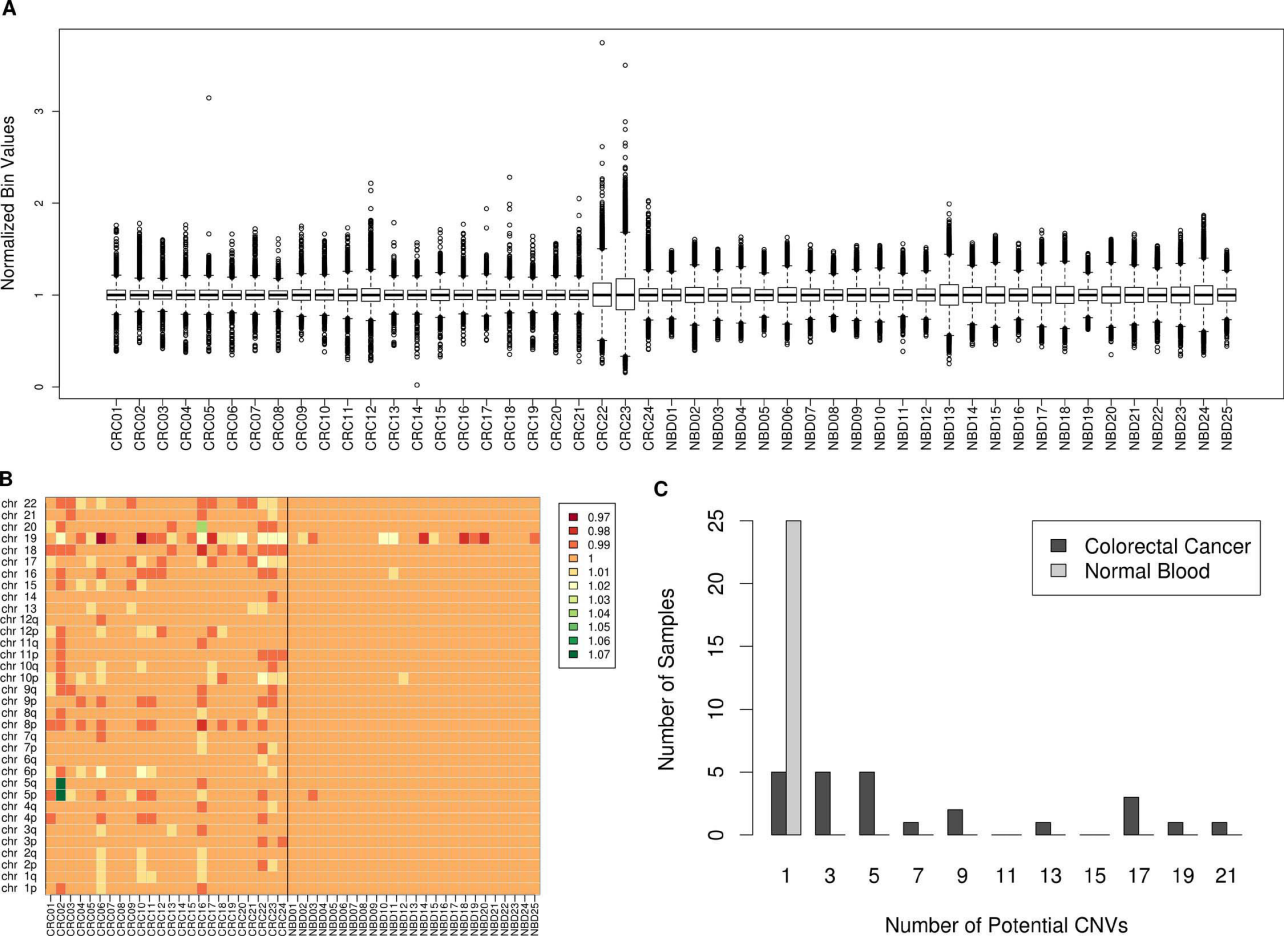
Cell free DNA CNV detection. A) Distribution of normalized 10Kb bin values in cfDNA in each cancer and normal blood sample. B) Heatmap of the genome broken down in large chromosomal segments colored based on their median normalized bin value. A value of 1 represents a normal diploid segment, values less than one are potential deletions, and values higher than one are potential amplifications. C) Distribution of the number of potential CNVs detected in normal vs colorectal cancer cfDNA samples.

We recruited 25 colorectal cancer (CRC) patients at time of first diagnosis of disease presenting at various stages (Stage 1-4b; Table 1). Circulating free DNA from these cancer samples along with 25 normal blood samples were processed to normalize read counts across the genome. CRC Sample CR025 failed to produce enough sequencing reads for processing and was dropped from further analysis. Briefly, the genome was divided into adjacent 10Kb bins and read counts were generated per bin. We removed any outlier bins, and then normalized the counts based on GC content of the bin, see Methods for more details. We have previously shown that a 1 copy gain or loss at 1% ctDNA can be detected with a 3X sequencing depth if the CNV is 30Mb or larger [7]. Therefore, in this study, we focused on CNVs affecting entire chromosomes for smaller chromosomes (chr13-chr22) or chromosome arm level changes for larger chromosomes (chr1-chr12) by merging the smaller 10Kb bins together. To detect regions with CNVs, we first normalized each of the 10Kb cancer sample bin counts to the median read count within the corresponding bin across all normal samples. Normal samples were also normalized this way, but without inclusion of the normal sample being normalized while calculating the median read count. The resulting normalized 10Kb bin counts were centered on 1 (Fig 1A) and there was no significant difference in the overall variance in normalized bin-count between CRC and normal samples (p-value = 0.18, Two-sided t-test), though some individual samples showed increased variance.

**Table 1:** Colorectal cancer samples staging, size, and metastasis.

ID	Evidence of Metastasis?	Tumor Cell Type	Tumor Histologic Grade	Margins	Size	T Stage	N Stage	M Stage	POST OP Colon CA Stage
**CR001**	Yes	Adenocarcinoma	G3 poorly differentiated	Negative	3.2cm x 5.0 cm x 0.7cm	T3	N2b	MX	Stage 3c
**CR002**	No	Tubular Adenoma	G2 moderately differentiated	Negative	7.0cm x 4.0cm x 2.4cm	T3	N0	Unknown	Stage 2a
**CR003**	Unknown or not specified	Adenocarcinoma	G2 moderately differentiated	Negative	3.0cm x 2.5 cm x 1.3cm	T4a	N2a	Unknown	Stage 3c
**CR004**	No	Adenocarcinoma	G1 well differentiated	Negative	3.0cm x 3.5cm x 1.0cm	T3	N1a	Unknown	Stage 3b
**CR005**	No	Adenocarcinoma	G2 moderately differentiated	Negative	2.5 cm x1.5cm x 1.0cm	T3	N1	Unknown	Stage 3b
**CR006**	No	Adenocarcinoma	G2 moderately differentiated	Negative	5.0cm x 4.5cm x 0.4cm	T2	N0	Unknown	Stage 1
**CR007**	No	Adenocarcinoma	G2 moderately differentiated	Negative	2.4cm x 1.8cm x 0.4cm	T1	N0	Unknown	Stage 1
**CR008**	No	Villous Adenoma		Negative	4.0cm x 3.0 cm x 1.7cm	T0	N0	M0	Stage 0 High Risk Adenoma
**CR009**	No	Adenocarcinoma	G2 moderately differentiated	Negative	4.4cm x 3.5cm x 0.7cm	T2	N0	Unknown	Stage 1
**CR010**	No	Mucinous Adenocarcinoma	G3 poorly differentiated	Negative	8.9cm x 5.0cm x 1.5cm	T3	N0	Unknown	Stage 2a
**CR011**	No	Invasive colonic carcinoma with focal mucin production	G2 moderately differentiated	Negative	7.0cm x 4.5cm x 1.0cm	T3	N0	Unknown	Stage 2a
**CR012**	No	Adenocarcinoma	G2 moderately differentiated	Negative	4.0cm x 3.8cm x 1.0cm	T3	N0	Unknown	Stage 2a
**CR013**	Unknown or not specified	Tubular adenoma	G2 moderately differentiated	Negative	Cecal tumor 5cm x 4cm x 1cm / Ascending tumor 2.2cm x 2cm x 1cm	T2	N0	M0	Stage 1
**CR014**	Yes	Mucinous Adenocarcinoma	G2 moderately differentiated	Negative	5.5cm x 6.3cm x 1.7cm	T4a	N2b	Unknown	Stage 3c
**CR015**	Unknown or not specified	Adenocarcinoma	G2 moderately differentiated	Negative	4.8cm x 3.8cm x 1.1cm	T3	N0	Unknown	Stage 2a
**CR016**	No	Invasive colonic adenocarcinoma	G2 moderately differentiated	Negative	10.8cm x 8.2cm x 5.0cm	T4b	N0	Unknown	Stage 3c
**CR017**	No	Mucinous Adenocarcinoma	G1 well differentiated	Negative	1.5cm x 1.3cm x 0.8cm	T2	N0	Unknown	Stage 1
**CR018**	Yes	Signet Ring Cell carcinoma	G3 poorly differentiated	Positive	7.8cm x 5.3cm x 1.5cm	T4a	N2b	M1b	Stage 4b
**CR019**	Yes	Adenocarcinoma	G3 poorly differentiated	Positive	1.8cm x 1.7cm x 0.8cm	T4a	N1c	Unknown	Stage 3b
**CR020**	Unknown or not specified	Tubulovillous adenoma	G2 moderately differentiated	Negative	9.0cm x 6.5cm x 1.4cm	T4a	N2a	Unknown	Stage 3c
**CR021**	Unknown or not specified	Adenocarcinoma	G2 moderately differentiated	Negative	3.1cm x 2.8cm x 1.5cm	T3	N1b	Unknown	Stage 3b
**CR022**	Unknown or not specified	Adenocarcinoma	G2 moderately differentiated	Negative	2.0cm x 1.8cm x 0.5cm	T1	N1a	Unknown	Stage 3a
**CR023**	Unknown or not specified	Adenocarcinoma	G2 moderately differentiated	Negative	2.0cm x 0.8cm x 0.8cm	T3	N0	Unknown	Stage 2a
**CR024**	Unknown or not specified	Adenocarcinoma	G2 moderately differentiated	Negative	4.0cm x 3.0cm x 0.9cm	T4a	N1a	Unknown	Stage 3b
**CR025**	No	Mucinous Adenocarcinoma	G1 well differentiated	Negative	1.5cm x 1.3cm x 0.8cm	T2	N0	Unknown	Stage 1

The normalized bins were then merged by taking the median of all the normalized values. This results in a value centered around 1.0 for each combined chromosomal segment, this means that a 1 copy change in the segment at 1% ctDNA burden will lead to, on average, a 0.01 shift from 1.0. We therefore considered every segment above 1.01 as a potential amplification and every segment below 0.99 as a region containing a potential deletion. On average, we detected a CNV in 8 ± 6.65 chromosomal segments out of 34 total segments in the CRC samples. On the other hand, normal samples only had 1 ± 0.65 segment with a CNV on average (p-value = 5.54E-07, Mann-Whitney U test), Fig 1C. Chromosome 19 was the most variable chromosome in terms of read counts and accounted for greater than 2/3rds of the CNVs in normal samples. In fact, if we disregard chr19, the average CNV per normal sample falls to nearly 0. The difference in number of CNVs detected can be used to distinguish cancer and normal samples. Using a cutoff of 2 CNVs or higher per sample, we could distinguish cancer samples from normal samples with an overall accuracy of 89.8% (95% confidence interval: 77.8% - 96.6%) and a 100% positive predictive value (PPV) (lower bound confidence interval: 82.4%), Table 2. If we disregard chr19 from our sensitivity analysis, we can drop our CNV cutoff to 1 or higher for calling a sample cancer. This way, we achieve the same results as before (Accuracy = 89.8%, PPV = 100%). Furthermore, the detected CNVs in the CRC samples lie in similar regions across samples, implicating an underlying cancer mechanism and not just random noise. The most commonly aberrant segments which are deleted in multiple CRC samples are chromosome 8p, 18, and 9p, while chromosome 6p, and 10p harbor frequent amplifications (Fig 1B). All 3 segments that we found to be frequently deleted in our samples are known to harbor CRC specific gene deletions as determined in a pan-cancer study [9]. Chromosome 6p was found to contain CRC specific amplifications, and although chromosome 10p amplification wasn’t found to be CRC specific, it is one of the prominent regions containing amplifications across all cancers. Chromosomes 8p and 18 were also in the top 3 segments deemed important for prediction of cancer by our previously reported random forest model [7]. Additionally, the most important segment for CRC prediction, chromosome 20, harbored frequent CNVs in our current cancer samples, but could either be an amplification or a deletion similar to previous findings where chromosome 20 is generally amplified in CRC, but can also have prominent deletions in the 20p12.1 region [9].

**Table 2:** Colorectal cancer prediction results.

	Normal Blood	All (n = 24)	CRC by Stage				
			0 (n = 1)	1 (n = 5)	2 (n = 6)	3 (n = 11)	4 (n = 1)
**Predicted Cancer**	0	19	0	4	5	9	1
**Predicted Normal**	25	5	1	1	1	2	0

Prediction of colorectal cancer based on ctDNA derived CNVs. Stage here is the post-op stage group of the tumor biopsy sample. Stage 0 represents high risk adenoma.

**Fig 2:**
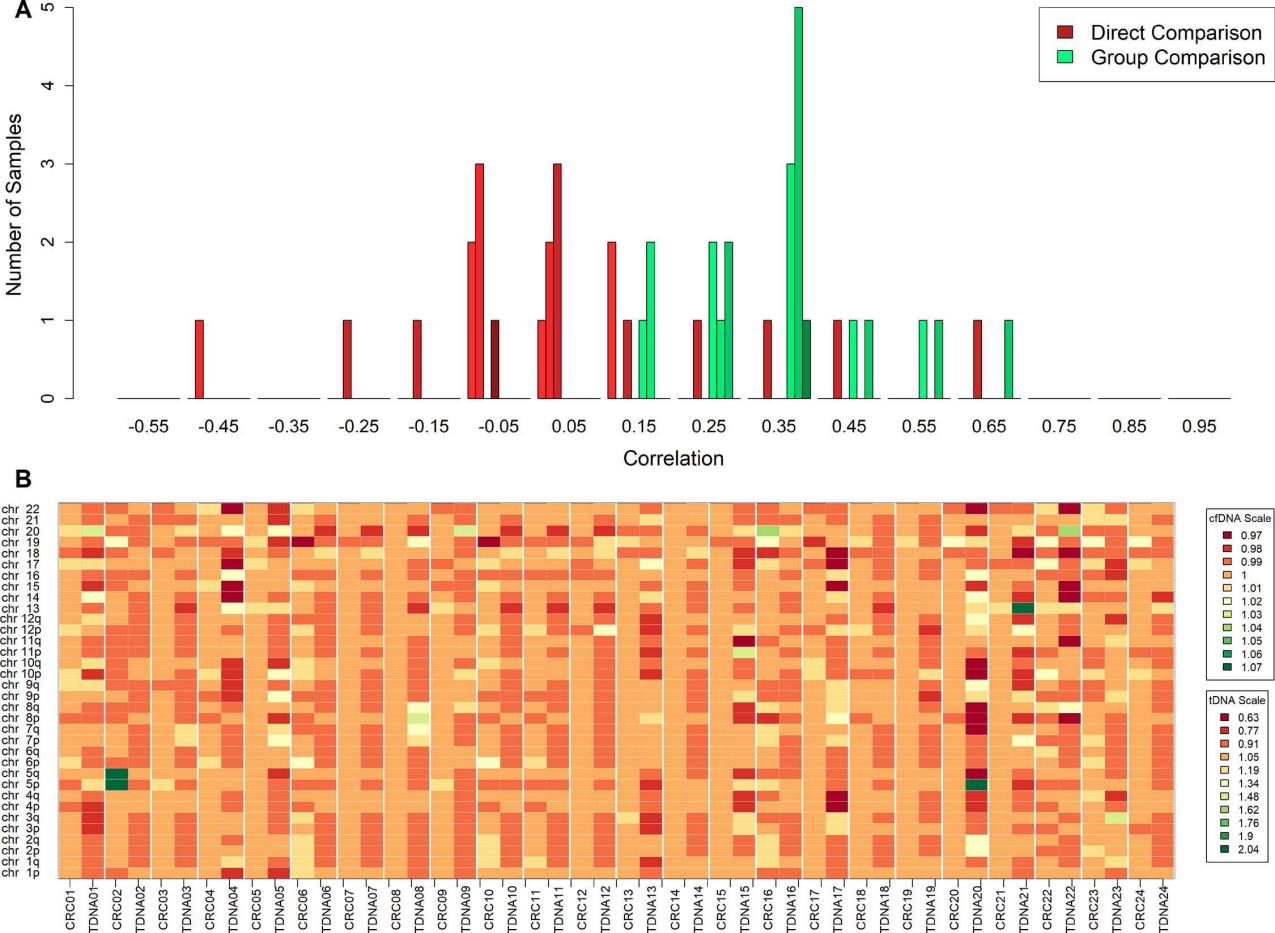
Comparison of cfDNA CNVs and tumor tissue CNVs. A) Distribution of direct correlation between CNVs detected via cfDNA samples and CNVs identified in parent tumor tissue (Red); darker color represents higher stage (1–4). Distribution of highest correlation between CNVs detected via a cfDNA sample when compared to all tumor tissue samples (Green); darker color represents higher stage (1–4). B) Heatmap of potential CNVs detected via cfDNA sequencing along with CNVs identified in parent tumor tissue scaled separately. A value of 1 represents a normal diploid segment, values less than one are potential deletions, and values higher than one are potential amplifications.

In addition, we carried out low pass whole genome sequencing of parent tumor tissue for each cfDNA sample, and used the data to call CNVs. CNVs in tumor tissue samples were called using the same method described for cfDNA, but instead of normalizing using normal-tissue samples, tumor tissue samples were normalized by the median read count of every tumor tissue sample other than the one being normalized. When CNVs detected via cfDNA were compared to the corresponding tissue CNVs, there was lack of any overall significant concordance, most cfDNA samples had near 0 correlation with CNVs in their matching tissue samples, 7 had positive correlation, some quite strong positive correlations, while the cfDNA profiles of 3 samples were negatively correlated with the corresponding tissue profile (Fig 2A–Direct Comparison). We hypothesize that the low concordance during direct comparison of cfDNA CNVs with tissue CNVs is a result of the characterization of sub-clonal CNVs detected via tissue biopsy. Cancer tissue biopsies collected were from a small region and probably failed to capture the full complexity detected via blood. In fact, it can be seen in Fig 2B that CNVs detected via cfDNA sequencing lie in regions similar to tissue CNVs if only in samples other than the parent tissue for example, TDNA20 has a similar chr5 gain as CRC02. To quantify this effect, we used all tissue samples as surrogate “clones” in the absence of repeated samplings from the same tumor tissue. We calculated correlations of each cfDNA sample to every tissue sample and selected the maximum value. This maximum value, in theory, would be similar to matching a cfDNA sample with the most prominent sub-clone present in the tissue. Using this approach, all cfDNA samples were positively correlated (Fig 2A–Group Comparison) to at least one other tissue sample suggesting that the CNV signal we detect via cfDNA largely resembles CRC CNV signal.

## Discussion

In this study, we have shown that detection of large somatic copy number alterations is feasible via low pass sequencing of circulating DNA at both early and late stages of colorectal cancer. A key component of low pass sequencing data analysis is noise removal. With our outlier removal using robust statistical estimates and normalization techniques, we were able to detect CNVs throughout the genome in CRC samples, with few potentially false positive signals in the normal samples, mostly limited to chr19. Large CNVs are generally not expected in normal blood samples [10]. It has been shown that genome wide nucleosome footprints can be recreated using cfDNA sequencing and that stable cfDNA is most likely histone bound [11]. This results in less DNA sequences being present in the blood for regions of the genome that are gene rich as these regions are more frequently transcribed resulting in constant disruption and replacement of histone molecules. Since chr19 is the most gene dense chromosome, our observation of fewer overall reads aligning to this region and a higher variance compared to all other chromosomes is potentially due to the effect of variable histone occupancy. Therefore, it might be beneficial to consider restricting analysis to gene sparse regions.

Out of the 24 CRC samples, we could detect more than 1 CNV in 19 of them and consequently were able to distinguish them from normal samples. These most prominent CNVs we detected lie in regions containing functionally important genes, for example the segments we observed to be deleted the most contain cell cycle related genes like CDKNA2, CDKN2B, and tumor suppressors like SMAD4. While the commonly amplified regions contain genes associated with angiogenesis like VEGF, and cyclin D3 which induces progression through G1 phase and promotes cell division. 3 of the 5 CRC samples that were undistinguishable from normal samples were Stage 2 or less (CR07, CR08, and CR15), while the other two were Stage 3b (CR19) and Stage 3c (CR14). It is possible that these samples simply had a very low ctDNA abundance or alternatively, the CNVs detected in the tissue were subclonal and below detection limits. Although ~43% colorectal cancers are known to be affected by whole genome duplication events [9], colorectal cancer has still been described as a primarily point mutation driven cancer [12], thus some variability in subclonal CNV profiles can be expected for CRC. The subclonal nature of CNVs in colorectal cancer can also be gauged by the lack of concordance we see between CNVs detected via cfDNA and parent tissue derived CNVs, but an overall high concordance when we compare cfDNA CNVs to CNVs from all tissue samples. While one would expect there to be a positive correlation between cfDNA and parent tissue CNVs, especially given that various studies detecting point mutations via cfDNA show high concordance with mutations in tissue samples [5, 6], it’s been recently shown that CNVs in CRC are highly discordant, even in the same primary tissue where they occupy distinct compartments while point mutations were more or less evenly distributed [13]. Given that our biopsy samples were only limited to single region of the tumor tissue, it is highly likely that our low concordance is due to sub-sampling of the tumor. Nevertheless, we attained 100% specificity and a sensitivity of 79% which outperforms other recently published methods for detecting early stage CRC [5, 6, 8]. However, we should note that our sample size was limited and might not be sufficient to gauge sensitivities with real world incidence rates.

CNVs play a major role in cancer etiology, and are the most common reason for genomic instability in CRC affecting 75–85% of tumors [14]. Currently, colorectal cancer is divided into any 4 different molecular-subtypes by the Colorectal Cancer Subtyping Consortium based on gene expression patterns and the presence or absence of genomic instability features like CNVs, microsatellite instability or CpG island methylation [15]. One can envision a future where classification of colorectal cancers is based on the presence of certain CNVs once we understand their molecular underpinnings and effects [16]. Furthermore, with the addition of clinical and lifestyle factors, early diagnoses of colorectal cancer can be further improved. With constant monitoring and careful examination, it might also be possible to link certain epidemiological factors directly to the emergence of somatic changes leading to adverse functional outcomes [17]. This sort of an analysis can help design proper interventions and clinical recommendations for the prevention of colorectal cancer [18].

CNVs are theoretically easier to detect than point mutations or epigenetic changes in a circulating DNA setting, and this pilot study clearly demonstrates the importance of CNV based cancer detection, and possibly monitoring and profiling. Nevertheless, we would like to point out that even though these patients were at different stages of their diseases, all of them still presented with symptoms and had resectable tumors, and it is possible that cfDNA CNV detection is limited to such cases. However, recent incidental findings of cancer in pregnant mothers during non-invasive prenatal testing [19] and the very high positive predictive value justifies the need for a larger trial with a cohort resembling actual incidence rates of cancer. Such a larger study with possibly more refined numerical/experimental noise removal and normalization methods will undoubtedly help advance early detection and monitoring of cancer in an affordable and reliable way.

## Materials and methods

### Sample collection and sequencing

Study participants provided written informed consent under the protocol entitled “Molecular Stethoscope for Colon Cancer Detection”, which was approved by the Scripps Institutional Review Board in 2015 (IRB-15-6616). The trial is also listed on clinicaltrials.gov (NCT02578264).

Subjects diagnosed with resectable colon cancer were contacted and consented to participate in the study. Subjects were required to not have undergone any chemo or radiation therapy. Blood samples were collected Pre-Op in Streck Tiger Top Cell free DNA and Pax Gene DNA tubes. Tumor tissue sample from the resected mass was collected and flash frozen in dry ice by pathologists for subsequent processing. Tumor staging, size, and other metadata generated by the pathologists was collected and documented.

Plasma from blood samples was isolated by centrifugation at 820g for 10 minutes, then a subsequent 10-minute centrifugation at 16000g to further reduce cellular contamination. ~10 ng/mL of cfDNA was isolated from plasma using Qiagen QIAamp Circulating Nucleic Acid kit. Tissue DNA was isolated from RNAlate stabilized tissue using Qiagen QIAamp DNA mini kit, and then tissue DNA was sheared to ~200bp using a Covaris ultrasonicator. 25ng isolated cfDNA or 25ng sheared tissue DNA was used as starting material for sequencing library prep. cfDNA and tissue DNA libraries were prepared using NEXTflex Dual-Indexed DNA Barcodes (Cat#514160, Illumina Compatible). The libraries were finally enriched by performing 3 cycles amplification using Kapa HiFi HotStart ReadyMix. Library concentrations were measured using Qubit, and library QC was done using Agilent Bioanalyzer. Pooled libraries were sequenced across 4 Illumina HiSeq2500 high throughput flow cells yielding enough sequences to get a 3X coverage of the genome per sample.

### Read processing

Sequenced Reads were processed to remove adapter contamination using Trimmomatic [20], duplicates reads were removed using Picard (http://broadinstitute.github.io/picard/) and finally, reads were aligned to the human genome build hg38 using the BWA-mem algorithm [21]. We divided the human genome into adjacent 10Kb bins filtering out any regions of the genome without GC content information or those that lie within the DAC blacklist regions, and counted read counts per bin using HTSeq-Count [22].

To account for genomic regions prone to mismapping and other biases, a Minimum Covariance Determinant estimator to determine a robust location (mean) and scale (variance) estimate of the expected read counts per bin [23]. Briefly, the MCD method looks for the h (>n/2) observations (out of n) whose classical covariance matrix has the lowest possible determinant. The estimate of location is then the average of these h points, whereas the estimate of scatter is their covariance matrix, multiplied by a consistency factor. To identify bins which consistently show up as outliers, we calculated the Mahalanobis distance for each genomic bin based on the raw estimate of the location and scatter and removed bins with a Mahalanobis distance greater than 1 standard deviation from the mean Mahalanobis distance. Out of the X total bins Y were marked as outliers using this approach. Finally, to correct for GC bias, we used a modified method proposed by Fan et. Al [24]. We fitted a LOESS curve to estimate the relationship between read count and GC content of the genomic bins for each sample separately. This relationship was utilized to normalize read counts for GC bias.

## Supporting information

S1 Table cfDNA yields. **c**fDNA yields for individual blood draws for each patient.
(XLSX)

S2 Table Numerical normalized CNV matrix. The final normalized and aggregated values for each chromosome level or chromosome arm level bins.
(XLSX)
